# Factors associated with late-stage presentation of cervical cancer in Ghana

**DOI:** 10.4314/gmj.v56i2.5

**Published:** 2022-06

**Authors:** Adu Appiah-Kubi, Thomas O Konney, Kwabena Amo-Antwi, Augustine Tawiah, Maxwell K Nti, Frank Ankobea-Kokroe, Sarah G Bell, Priscilla K Appiah-Kubi, Carolyn Johnston, Emma R Lawrence

**Affiliations:** 1 School of Medicine, Department of Obstetrics and Gynecology, University of Health and Allied Sciences, PMB 31, Ho, Volta Region, Ghana; 2 Department of Obstetrics and Gynaecology, Gynaecologic Oncology Unit, Komfo Anokye Teaching Hospital, Okomfo Anokye Road, Kumasi, Ghana; 3 Department of Obstetrics and Gynecology, School of Medicine and Dentistry, Kwame Nkrumah University of Science and Technology, Accra Road, Kumasi, Ghana; 4 Department of Obstetrics and Gynecology, University of Michigan, 1500 East Medical Center Drive, Ann Arbor, Michigan, USA; 5 Department of Pharmacy, Korle Bu Teaching Hospital, Guggisberg Avenue, Accra, Greater Accra Region, Ghana; 6 Division of Gynecologic Oncology, University of Michigan, 1500 East Medical Center Drive, Ann Arbor, Michigan, USA

**Keywords:** uterine cervical neoplasms, uterine cervical cancer, gynecologic neoplasm, Ghana

## Abstract

**Objective:**

To explore factors associated with late clinical presentation among Ghanaian women with cervical cancer

**Design:**

This is a cross-sectional survey using a paper questionnaire.

**Setting:**

Komfo Anokye Teaching Hospital (KATH) in Kumasi, Ghana.

**Participants:**

Participants were women presenting for cervical cancer care at KATH. Inclusion criteria were histologically diagnosed cervical cancer and age ≥18 years. The exclusion criteria was age <18. All women presenting from August 2018-August 2019 were recruited.

**Main outcome measures:**

The primary outcome was the proportion of participants presenting with late-stage cervical cancer, defined as stage II or higher.

**Results:**

Of 351 total participants, 33.6% were unemployed, 35.3% had no formal education, and 96.6% had an average monthly income of less than five hundred Ghana cedis ($86 USD). Time from symptoms to seeing a doctor ranged from fewer than two weeks (16.0%) to more than twelve months (8.6%). Participants' most common barrier in seeking healthcare was financial constraints (50.0%). Most participants presented at late-stage cervical cancer (95.2%, n=334), with only 4.8% (n=17) presenting at stage I. Of participants presenting with late-stage cervical cancer, the vast majority had never had a Papanicolaou (Pap) smear (99.1%) nor a recent gynecologic exam (99.3%). After adjusting for age, parity, and distance to a healthcare facility, a late-stage presentation was associated with lower income and living in a rural area.

**Conclusions:**

In Ghana, 95% of women with cervical cancer seek care at a late clinical stage, defined as stage II or greater, when the cancer is inoperable.

**Funding:**

None declared

## Introduction

Cervical cancer is the fourth most common cancer in women worldwide.^1^, ^2^ There have been 528,000 newly diagnosed cases and 266,000 mortalities in the last ten years.^1^ Human papillomavirus (HPV) is the main causative organism in cervical cancer; however, there are significant co-factors, including high parity, tobacco use, immunosuppression, and low socioeconomic status.^3^, ^4^

In high-income countries, screening programs for cervical pre-cancer and HPV have considerably decreased the prevalence of cervical cancer.^5^ Additionally, HPV vaccination programs have shown promise in reducing long-term cervical cancer rates.

Low- and middle-income countries have an undue burden of cervical cancer.^7^ An estimated 85% of newly diagnosed cases of cervical cancer, and almost nine of 10 mortalities, occur in low-resource settings.^7^ In Sub-Saharan Africa, 34.8 new cases of cervical cancer are diagnosed per 100,000 women annually, with a mortality rate of 22.5 per 100,000 women. This compares with an incidence of 6.6 and a mortality rate of 2.5 per 100,000 women in North America.^2^ Cervical cancer is one of the leading causes of cancer-related death among women in Ghana, and the incidence and mortality rate is among the highest worldwide.^8^ In 2018, cervical cancer was Ghana's most common gynecologic cancer, with an estimated 3,151 newly diagnosed cases and 2,119 deaths.^6^ Significant variations in incidence and mortality rates between low- and high-resource settings may be explained by the lack of access to effective screening and services that expedite early detection and treatment.^9^ This is compounded by cultural attitudes and inadequate public education, which hamper early diagnosis and treatment.^9^

Cervical cancer spreads by direct invasion of the surrounding tissues, extension through lymphatic channels to regional lymph nodes, and hematologic metastasis to the liver, lungs, and skeleton.^10^ Early stages of cervical cancer, defined as International Federation of Gynaecology and Obstetrics (FIGO) stage IA, IB1 and 1B2, are surgically treated with conization or hysterectomy.^11^ Late-stage cervical cancer, defined as FIGO stage II or greater, is generally considered inoperative and is managed with chemotherapy and radiation.^12^ Prognosis varies dramatically based on the stage at presentation, with five-year survival rates of 95% for stage I, 70% for stage II, 40% for stage III, and only 15% for stage IV.^13^, ^14^ Management of late-stage cervical cancer is particularly difficult in low-resource settings, where affordability and access to chemotherapy and radiation are limited.

In many low-resource settings, including Ghana, women with cervical cancer often delay presentation for healthcare until the disease is at a late stage and is often incurable, making treatment very difficult.^15^ An older study in Ghana conducted by Dunyo et al. among 147 women demonstrated that two-thirds of cervical cancer patients presented at a late stage (defined as stage II or higher).^28^ A study in Uganda demonstrated that 61% of cervical cancer patients presented at a late stage (defined as stage III or IV in the former FIGO staging criteria),^16^ and a study in Nigeria demonstrated that 89% of cervical cancer patients presented at a late stage (defined as stage IIB or higher in the former FIGO staging criteria).^17^

Limited research has been conducted in women who present with cervical cancer to understand the initial actions taken after first noticing symptoms and the predictors of late presentation to care. This current study addresses this question by exploring the nature of presentation and factors associated with late clinical presentation among women with cervical cancer in Kumasi, Ghana. Gaining a better understanding of predictors of late presentation will help develop and target patient counselling and public health campaigns to promote the earlier presentation of cervical cancer among at-risk women.

## Methods

### Study design

This cross-sectional study uses a survey design to explore factors associated with late clinical presentation among women with cervical cancer.

### Setting

This study was conducted at the specialist gynecologic oncology clinic at the Komfo Anokye Teaching Hospital—an urban tertiary care hospital located in Kumasi, Ghana. The hospital's Department of Obstetrics and Gynecology includes a gynecologic oncology unit, one of Ghana's largest cervical cancer treatment centres. It is staffed by two gynecologic oncologists and five fellows in training. The unit manages patients from across Ghana and neighbouring West African countries. Cervical cancer care, the unit provides includes diagnosis, surgical management, and coordination of chemotherapy and radiotherapy. National guidelines in Ghana recommend screening for cervical cancer with visual inspection with acetic acid for women aged 25–45 years and cytology screening with Papanicolaou smear for women aged 45 and above. However, Ghana lacks an effective national screening program, and rates of cervical cancer screening in Ghana and at KATH are low.^18^ Although routine gynaecology visits are covered by Ghana's National Health Insurance Scheme, cervical cancer screening and pathologic diagnosis of cervical cancer are not covered.

### Participants

Participants were women presenting for treatment for cervical cancer at the specialist gynecologic oncology clinic at Komfo Anokye Teaching Hospital. Inclusion criteria were new presentation for cervical cancer care at the hospital during the study period, histologically diagnosed cervical cancer, and age ≥18 years. All participants were newly diagnosed with cervical cancer at KATH; no participants were referred with a pre-existing diagnosis of known cervical cancer. Data were collected for 12 months, from August 2018-August 2019. Kwame Nkrumah University of Science and Technology, School of Medical Sciences/KATH (CHRPE/AP/456/18) granted Institutional Review Board approval.

### Data sources and variables

After written informed consent was obtained, a three-part structured questionnaire was administered to each study participant by a trained research assistant in either English or a local dialect (Twi), depending on the participant's language preference. All questions were in a multiple choice format with categorical responses. Section one focused on demographic characteristics, including age, marital status, urban versus rural residence, ethnicity, and religion. Demographic characteristics, including urban versus rural residence, were self-defined by participants. Socioeconomic factors included occupation, income, level of completed education, and distance to the nearest health facility. The background health questions included the presence of comorbidities such as hypertension and diabetes, human immunodeficiency virus status, parity, number of lifetime sexual partners, and family history of cancer. Section two focused on perceived knowledge about cervical cancer, including awareness of cervical cancer and modes of hearing about cervical cancer, as well as awareness of cervical cancer screening programs, screening modalities, and treatment options. Finally, participants were asked about the symptoms that prompted them to seek medical care, Pap smear screening, and a gynecologic examination in the past three years. Section three focused on reasons for the timing of presentation to any healthcare facility. Participants were asked who they informed about their symptoms, what advice was given, and the first action they took when symptoms started. They indicated the time that elapsed from symptoms to seeking care from a doctor and the type of health facility where they first sought care. Finally, they indicated all barriers to seeking care from a provided list. Clinical information on the stage of cervical cancer patients presented to the clinic was obtained from participants' medical records. As part of standard clinical care, all participants had been clinically staged according to FIGO staging by their gynecologic oncologist. The late-stage presentation was defined as Stage II or greater.

### Sample size

A sample size of 350 participants would provide 80% power to detect a 7% difference in late presentation of cervical cancer, assuming two-sided tests and an alpha of 0.05. Sample size calculations were performed using STATA.

### Data Analysis

Analysis was carried out using STATA version 15.0 (Stata Corporation, Texas, USA). Frequencies and proportions were used to describe categorical variables, and medians and ranges were used to describe non-normally distributed continuous variables. For analysis, single, divorced, widowed, and separated women were grouped as “non-married” due to the low numbers of participants in each sub-group. Bivariate analysis using T-tests for continuous variables and Chi-squared and Fischer's Exact for categorical variables was used to compare demographic variables and health-seeking behaviours across early-stage versus late-stage presentation of cervical cancer. Variables that were significant in the bivariate model at p < 0.05 were included in a final logistic regression model to evaluate predictors of late-stage presentation of cervical cancer. The regression was also adjusted for variables the authors felt were clinically important, including parity, having undergone a gynaecology examination in the past three years, and distance to a healthcare facility. Results were reported using adjusted odds ratios and 95% confidence intervals. P-values <0.05 were considered statistically significant.

## Results

During the study period, 950 patients were seen in the gynecologic oncology unit at Komfo Anokye Teaching Hospital; of these, 370 were diagnosed with cervical cancer, 360 met all eligibility criteria, and nine were eligible candidates who declined participation. A total of 351 participants were recruited and included in the analysis.

Most participants were ≥66 years old (n=134, 38.2%) with a range from 32 to 87 years, Christian religion (n=315, 89.7%), and Ashanti ethnicity (n=217, 62.0%) (Table 1). Approximately half of the participants were unmarried (n=194, 55.3%), defined as divorced, widowed, or never married. Regarding socioeconomic status, 33.6% (n=118) of participants were unemployed, and if employed, the most common occupation was being a trader (n=142, 40.5%). Most participants had an average monthly income of <500 cedis (n=339, 96.6%), in the context of Ghana's minimum wage of 355 cedis. Half lived in a rural area (n=179, 51.0%), and 35.3% (n=124) did not have any formal education. Most participants' distance to the nearest health facility was <10 kilometers (n=343, 97.7%). Regarding their relevant health status, the median parity was 4 (range 0–13), 25.1% (n=88) had a comorbid chronic condition, 0.6% (n=2) were positive for the human immunodeficiency virus, and 15.7% (n=55) had a family history of any cancer.

**Table 1:** Demographics of participants

Variable	n (%) or mean ± standard deviation
**Age, years**	
**26–35**	16 (4.6)
**36–45**	36 (10.3)
**46–55**	93 (26.5)
**56–65**	72 (20.5)
**≥66**	134 (38.2)
**Marital status**	
**Married**	157 (44.7)
**Not married**	194 (55.3)
**Religion**	
**Christian**	315 (89.7)
**Muslim**	34 (9.7)
**Traditionalist**	2 (0.6)
**Employment status**	
**Employed**	228 (65.0)
**Not currently employed**	123 (35.0)
**Education level**	
**No formal education**	124 (35.3)
**Primary**	70 (20.0)
**Junior high school**	58 (16.5)
**Secondary high school**	35 (10.0)
**Tertiary**	14 (4.0)
**Other**	50 (14.3)
**Average monthly income,** **Ghana cedis**	
**<500**	339 (96.6)
**500–749**	2 (0.6)
**750–999**	3 (0.9)
**1000–1249**	4 (1.1)
**≥1250**	3 (0.9)
**Location of residence**	
**Urban**	172 (49.0)
**Rural**	179 (51.0)
**Distance to nearest health facility,** **kilometers**	
**<3**	144 (41.0)
**3–6**	154 (43.9)
**7–10**	45 (12.9)
**>10**	8 (2.3)
**Parity**	4 (IQR 3–6; Range 0–13)

**Figure 1:**
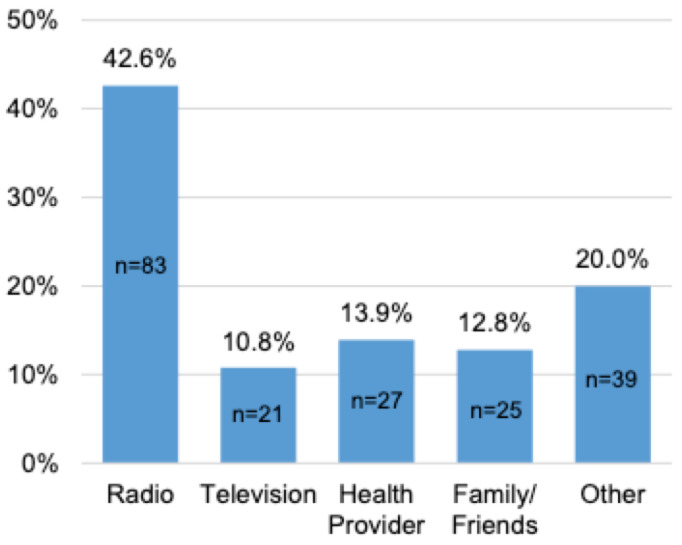
Awareness of cervical cancer

Fifty-five per cent (n=195) of participants reported general awareness about cervical cancer, while 44.4% (n=156) had never heard of cervical cancer. Of participants who were generally aware of cervical cancer, the most common source of information was the radio (n=83, 42.6%) (Figure 1).

**Figure 2:**
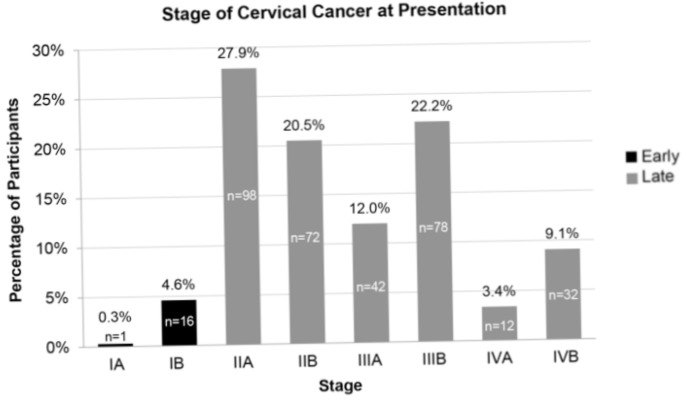
Presentation at early stage (I) versus late stage (II-IV)

Compared to general awareness, fewer participants were aware of specific aspects of cervical cancer, including screening and treatment. Of the 195 participants who had a general awareness about cervical cancer, only 3.6% had an awareness of screening programs for cervical cancer, 3.6% had an awareness of the Pap smear as a screening strategy, and 2.1% had awareness about treatments for cervical cancer. Most participants presented at stage II (n=170, 48.4%), with only 4.9% (n=17) presenting at Stage I (Figure 2).

The most common symptom that prompted presentation was postmenopausal bleeding (n=151, 43.0%), followed by vaginal discharge (n=115, 32.8%) (Table 2). The vast majority had never had a Pap smear (n=348, 99.2%) nor a recent gynecologic exam (n=317, 90.3%).

**Table 2:** Characteristics of presentation with cervical cancer

Variable	n (%)
**Symptom that prompted presentation to care** **Intermenstrual bleeding** **Postmenopausal bleeding** **Post-coital bleeding** **Pelvic pain** **Vaginal discharge** **Other**	24 (6.8%) 151 (43.0%) 3 (0.9%) 25 (7.1%) 115 (32.8%) 33 (9.4%)
**Pap smear history** **Never / unsure** **More than 3 years ago** **During the last 3 years**	348 (99.2%) 2 (0.6%) 1 (0.3%)
**Gynecology exam history** **No exam in last 3 years** **Exam in last 3 years**	317 (90.3%) 34 (9.7%)
**Person first informed of symptoms** **Husband** **Relative** **Religious leader** **Traditional healer** **Healthcare worker** **Other**	110 (31.3%) 103 (29.3%) 29 (8.3%) 11 (3.1%) 84 (23.9%) 14 (4.0%)
**Advice given by informant about symptoms** **Go to health facility** **Go to traditional healer** **Buy medication from pharmacy** **Use herbal medication** **Go to religious center** **Do nothing** **Other**	321 (91.5%) 2 (0.6%) 1 (0.3%) 5 (1.4%) 3 (0.9%) 1 (0.3%) 18 (5.1%)
**Action taken based on symptoms** **Went to health facility** **Went to traditional healer** **Used medication from pharmacy** **Used herbal medication** **Went to religious center** **Did nothing** **Other**	330 (94.0%) 10 (2.9%) 1 (0.3%) 1 (0.3%) 0 (0.0%) 1 (0.3%) 8 (2.3%)
**Time from symptoms to seeing a doctor** **<2 weeks** **2–4 weeks** **>4 week to 3 months** **>3 months to 6 months** **>6 months to 12 months** **>12 months**	57 (16.3%) 90 (25.7%) 78 (22.3%) 71 (20.3%) 24 (6.9%) 30 (8.6%)
**Health facility first presented to** **Health center** **District Hospital** **Regional hospital** **Tertiary hospital** **Private hospital**	56 (16.0%) 240 (68.4%) 12 (3.4%) 10 (2.9%) 33 (9.4%)
**Main barrier faced to seeking care** **Financial constraints** **No health insurance** **Thought sickness was spiritual** **Fear of hospitals** **No transportation to hospital** **Other**	174 (50.0%) 16 (4.6%) 14 (4.0%) 13 (3.7%) 7 (2.0%) 124 (35.6%)

Women most frequently first informed their husbands, a relative, or a healthcare provider about their symptoms, and the majority were advised to go to a healthcare facility (n=321, 91.5%).

Most participants reported that the first action they took was to present to a healthcare facility (n=330, 94.0%), and most presented to a district hospital (n=240, 68.4%). Time from symptoms to seeing a doctor ranged from <2 weeks (n=56, 16.0%) to >12 months (n=30, 8.6%). The most common barrier participants faced in seeking healthcare was financial constraints (n=174, 50.0%).

Using bivariate regression, variables that emerged as significant predictors of late-stage presentation of cervical cancer were age, income level, rural versus urban residence, the symptom that prompted presentation to care, whether a gynecologic exam was done within the last three years, type of healthcare facility first presented to with symptoms (Table 3).

**Table 3:** Bivariate analysis of predictors of early stage versus late stage of cervical cancer

Characteristic 1		Early Stage	Late Stage	p value
**Age, years**	26–35	2 (11.8%)	14 (4.2%)	p = 0.028*
	36–45	4 (23.5%)	32 (9.6%)	
	46–55	1 (5.88%)	92 (27.5%)	
	56–65	6 (35.3%)	66 (19.8%)	
	≥66	4 (23.6%)	130 (38.9%)	
**Marital status **	Married	7 (41.2%)	150 (44.9%)	p = 0.763
	Not married	10 (58.8%)	184 (55.1%)	
**Employment status**	Employed	8 (47.1%)	220 (65.9%)	p = 0.113
	Unemployed	9 (52.9%)	114 (34.1%)	
**Education level**	No formal education	2 (16.7%)	122 (42.2%)	p = 0.62
	Primary	4 (33.3%)	66 (22.8%)	
	Junior high school	1 (8.3%)	57 (19.7%)	
	Secondary high school	3 (25.0%)	32 (11.1%)	
	Tertiary	2 (12.7%)	12 (4.2%)	
**Monthly income (Ghana cedis)**	<500	15 (88.2%)	324 (97.0%)	p < 0.001*
	500–749	0 (0.0%)	2 (0.6%)	
	750–999	0 (0.0%)	3 (0.9%)	
	1000–1249	0 (0.0%)	4 (1.2%)	
	≥1250	2 (11.9%)	1 (0.3%)	
**Location of residence**	Urban	14 (82.4%)	158 (47.3%)	p = 0.005*
	Rural	3 (17.7%)	176 (52.7%)	p = 0.622
**Distance to nearest health facility (km)**	< 3	6 (35.3%)	138 (64.7%)	
	3 or greater	11 (64.7%)	196 (58.7%)	
**Parity** 2		4.6	4.8	p = 0.852
**Symptom that prompted presentation** **to care**	Intermenstrual bleeding	4 (23.5%)	20 (6.0%)	p = 0.014*
	Postmenopausal bleeding	9 (52.9%)	142 (42.5%)	
	Post-coital bleeding	0 (0.0%)	3 (0.9%)	
	Pelvic pain	2 (11.8%)	23 (6.9%)	
	Vaginal discharge	0 (0.0%)	115 (32.8%)	
**Pap smear history**	Never done / unsure	17 (100.0%)	331 (99.1%)	p = 0.926
	Done >3 years ago	0 (0.0%)	2 (0.6%)	
	Done within last 3 years	0 (0.0%)	1 (0.3%)	
**Gynecologic exam done within last 3** **years**	Not done	13 (46.5%)	304 (91.3%)	p = 0.048*
	Done	4 (23.5%)	30 (8.9%)	
**Person first informed of symptoms**	Husband	6 (35.3%)	104 (31.1%)	p = 0.595
	Relative	7 (41.2%)	96 (29.7%)	
	Religious leader	0 (0.0%)	29 (8.7%)	
	Traditional healer	0 (0.0%)	11 (3.3%)	
	Healthcare worker	4 (23.5%)	80 (234.0%)	
**Time from symptoms to seeing a doctor**	<2 weeks	5 (29.4%)	52 (16.3%)	p = 0.418
	2 to 4 weeks	4 (23.5%)	86 (25.8%)	
	>4 weeks to 3 months	1 (5.9%)	77 (23.1%)	
	>3 months to 6 months	3(17.7%)	68 (20.4%)	
	>6 months to 12 months	2 (11.8%)	22 (6.6%)	
	> 12 months	2 (11.8%)	28 (8.4%)	
**Health facility first presented to**	Health center	2 (11.8%)	54 (16.2%)	p <0.001*
	District hospital	3 (17.7%)	237 (71.0%)	
	Regional hospital	1 (5.9%)	11 (3.3%)	
	Tertiary hospital	3 (17.7%)	7 (2.1%)	
	Private hospital	8 (47.1%)	25 (7.5%)	

1 Comparison made with Chi-squared or Fisher's Exact unless otherwise indicated

2 Comparison made with t-test

* significant at p <0.05

In addition to factors that were significant in the bivariate comparisons, the final logistic regression model was also adjusted for parity and distance to a healthcare facility (Table 4).

**Table 4:** Predictors of presentation at a late stage of cervical cancer: final regression model

Characteristic	Adjusted Odds Ratio	95% Confidence Interval	P value^a^
**Age**	1.15	0.67–1.98	0.61
**Parity**	1.19	0.86–1.65	0.29
**Income level**	0.49	0.25–0.94	0.03*
**Education level**	1.04	0.58–1.86	0.90
**Rural (vs urban)**	11.53	1.34–98.80	0.03*
**Gynecology exam in last 3 years (vs none)**	0.77	0.13–4.68	0.78
**Healthcare facility <3km away (versus** **≥3km away)**	4.3	0.90–20.55	0.07

* Significant at p=0.05

Type of healthcare facility was associated with place of residence, with a higher proportion of women in urban areas first seeking care at the tertiary hospital and private hospitals compared to rural women. The type of healthcare facility was dropped from the final regression model due to collinearity. Each increasing level of income was associated with a 51% decrease in late-stage presentation (odds ratio 0.49, 95% CI 0.25–0.94, p=0.03). Compared to residence in an urban area, living in a rural area is associated with 11-times increased odds of late-stage presentation (odds ratio 11.5, 95% CI 1.34–98.80, p=0.26).

## Discussion

Only sixty per cent of all respondents in our study were aware of cervical cancer prior to their own diagnosis. The vast majority had never had a Pap smear nor a recent gynecologic exam. The most common barrier participants faced in seeking healthcare was financial constraints. Ninety-five per cent of participants presented at a late stage of cervical cancer, at which point their cancer was inoperable. Factors associated with the late presentation of cervical cancer are lower income and living in a rural area.

Despite the fact that cervical cancer is the fourth most common cancer worldwide,^1^, ^2^ global knowledge and awareness of cervical cancer among women is known to be low. Even in higher-income countries, many women are unaware of the typical symptoms and risk factors for cervical cancer.^19^–^20^ In Africa, where cervical cancer remains a serious public health issue, our findings are consistent with research done in Cameroon demonstrating that 68% of women had never heard of cervical cancer.^21^ Poor knowledge among women was also demonstrated in studies in Ethiopia,^22^ Nigeria,^23^ South Africa,^24^ and Kenya.^25^ In low-resource settings, older age, higher education, and higher income were significantly associated with higher knowledge about cervical cancer.^26^–^27^

Our study demonstrated that most women heard about cervical cancer from radio and television (43%), healthcare providers (14%), and family and friends (13%).

This is comparable to a study done in Nigeria, in which the most common source of information on cervical cancer was the media, followed by healthcare workers, schools, the internet and posters.^21^ Basic awareness about cervical cancer is important because having knowledge about cervical cancer is associated with utilization of cervical cancer screening.^28^–^29^

Late-stage presentation is complex, with contributions from patient-level, healthcare facility-level, and systems-level factors. Patient level delays can be partially explained by low health literacy and poverty, with a lack of knowledge about where and when to seek care, and financial barriers to afford needed care.^30^ The majority of women in our study presented for care due to postmenopausal bleeding (43%) and vaginal discharge (33%), which is consistent with the literature.^31^ Educating women on the importance of seeking prompt care for these specific symptoms, and informing families that routine gynaecology evaluation for these symptoms is covered by national health insurance, may promote earlier care seeking. Healthcare facility-level delays may be explained by a lack of training, personnel, and equipment available to adequately assess and diagnose symptoms concerning cervical cancer, especially at non-tertiary care facilities. Although the majority of women in our study did seek care from a healthcare facility for their symptoms, less than 10% report undergoing a gynecologic exam. The majority of women first sought treatment at primary healthcare facilities. Thus, there is a need to equip healthcare providers at all levels with training and equipment to provide gynecologic exams in patients who present with symptoms concerning cervical cancer, in addition to counselling and referring patients. Finally, systems-level barriers include a lack of national screening programs and incomplete coverage of key services by national health insurance. Despite advances in screening for cervical cancer, a predominance in late-stage presentation persists—highlighting the importance of affordability and access to screening and diagnostic care. In Ghana, national insurance covers routine gynaecology exams, however, does not cover cervical cancer screening or pathologic diagnosis of cervical biopsies, which are essential to respectively prevent and confirm cervical cancer.

Consistent with our findings, living in rural areas has been reported in the literature as a barrier to the early presentation of cervical cancer.^32^ In other studies, socio-economic factors have also been associated with late presentation of cervical cancer, including lack of health insurance,^32^ lower income,^33^ a low level of education,^34^ and lower literacy.^35^ The relationship between socioeconomic factors and stage of presentation may be explained by trends in sexual behaviour including early onset of sexual intercourse and number of partners, as well as knowledge of cervical cancer and access to health services.

This study makes an important contribution to the literature by exploring factors that are associated with late clinical presentation among women with cervical cancer. The study population is composed of women with histologically confirmed cervical cancer, which is a focused, highest-risk group. Limitations include that some respondents first experienced symptoms more than 12 months prior to the survey, which may contribute to recall bias. Respondents were prospectively recruited and surveys were conducted at the time of diagnosis, thus limiting this bias. Surveys were administered following the patient's clinic visit with their gynecologic oncologist; thus, responses could be affected by respondents' emotional distress or physical pain secondary to their cervical cancer diagnosis. Since surveys were conducted in a healthcare setting, respondents could be less comfortable disclosing delays in seeking care or negative attitudes about hospitals. To minimize these biases, surveys were conducted by a research assistant who was not a part of the healthcare team and a comprehensive informed consent process was conducted.

This research was carried out at a single tertiary care hospital, which may limit generalizability. However, the capacity to care for patients with cervical cancer is limited in low- and middle-income countries, and the study site cares for women from across Ghana and surrounding West African countries. Data on referral status was not collected, nor was the duration of time between being seen at an initial healthcare centre and being seen at KATH; this information could have enhanced understanding of healthcare-seeking behaviours and systems-level delays in diagnosis. Notably, only patients who presented for hospital-based care, had a biopsy performed, and followed up to attend a clinical visit were recruited as participants in this study. Thus, the perspectives of women in the community who were unable to access care were not included. Presumably, these women have even less knowledge and awareness about cervical cancer and face even more significant barriers to presentation for care compared to our study population. Additional qualitative research is needed to build upon this study to explore the nuances of financial challenges in the context of health insurance, and the experiences of women at referral centres, prior to their presentation for care at a tertiary centre.

Based on the predictors of late presentation to care identified by this study, the following public health and policy recommendations should be considered. At a patient level, we identify that only 60% of women were aware of cervical cancer. Thus, women should be educated about the common symptoms of cervical cancer and the risks associated with delays in seeking care.

For reproductive-age women, frequent contact with healthcare providers is experienced during prenatal and family planning care. These are opportunities for women's health providers to educate women about future warning symptoms, and to provide cervical cancer screening. In addition, women should be educated on the importance of engaging in routine gynaecology care after pregnancy, and reassured that gynecologic exams are covered by national health insurance in Ghana. Women with risk factors for late presentation, including low-income and rural inhabitance, should be specifically targeted by educational campaigns. At a healthcare facility level, we identify that most women did present to a healthcare facility for their symptoms, however, 90% did not undergo a recent gynaecology exam, and only 2.9% initially presented to a tertiary level facility. Thus, providers at every level, especially in district hospitals and health centres, should be equipped with the training and equipment to provide gynecologic exams in patients who present with symptoms of cervical cancer. At a systems level, we identify that 99% of women had never been screened for cervical cancer, and the most common barrier to seeking healthcare was financial constraints. The incidence of all cervical cancer can be significantly decreased by developing a national cervical cancer screening program, which incorporates pap smear and visual inspection with acetic acid as locally appropriate. In order to promote earlier diagnosis of cervical cancer, key gynecologic services, including cervical biopsy and pathologic interpretation, should be affordable to all women and covered by national health insurance.

## Conclusion

In all settings, early diagnosis of cervical cancer is vital to successful management and survival. Management of late-stage cervical cancer is very challenging in low-resource settings; therefore, in most instances, treatment is palliative. At a high-volume gynaecology oncology centre in Ghana, we demonstrate that the vast majority of women with cervical cancer present to care at a late stage. In this population, awareness about cervical cancer and utilization of screening services and routine gynecologic care is low. Women living in rural areas and those with lower income were more likely to present at a late stage.
